# Successful Treatment of Relapsed/Refractory Extramedullary Multiple Myeloma With Anti-BCMA CAR-T Cell Therapy Followed by Haploidentical Hematopoietic Stem Cell Transplantation: A Case Report and a Review of the Contemporary Literature

**DOI:** 10.3389/fmed.2021.649824

**Published:** 2021-05-07

**Authors:** Ying Qian, Zijun Qian, Xiujie Zhao, Wenjue Pan, Xinzheng Wei, Huimin Meng, Lin Yang, Haowen Xiao

**Affiliations:** ^1^Department of Hematology, Sir Run Run Shaw Hospital, Zhejiang University School of Medicine, Hangzhou, China; ^2^Hangzhou Integrative Medicine Hospital, Hangzhou, China; ^3^PersonGen BioTherapeutics (Suzhou) Co., Ltd., Suzhou, China; ^4^State Key Laboratory of Radiation Medicine and Protection, Collaborative Innovation Center of Hematology, CyrusTang Medical Institute, Soochow University, Suzhou, China; ^5^Institute of Hematology, Zhejiang University, Hangzhou, China

**Keywords:** extramedullary multiple myeloma, chimeric antigen receptor T-cell, refractory, haploidentical allogeneic hematopoietic stem cell transplantation, multiple myeloma

## Abstract

Extramedullary multiple myeloma (EMM) is an aggressive sub-entity of multiple myeloma (MM). Despite an excellent improvement in survival for most patients with MM over recent decades, the overall survival (OS) of patients with EMM was usually not longer than 3 years. Standard treatment for patients with EMM has not been established, and their management is particularly challenging. We presented a heavily pretreated young patient with relapsed EMM and refractoriness to a proteasome inhibitor (PI; bortezomib), a next-generation PI (ixazomib), immunomodulatory drugs (IMiDs; lenalidomide), autologous hematopoietic stem cell transplantation (ASCT), and monoclonal antibody (directed against CD38: daratumumab) and indicated that myeloablative haploidentical hematopoietic stem cell transplantation (haploidentical-HSCT) as a salvage treatment of relapse after a chimeric antigen receptor (CAR)-T cell therapy that targeted B-cell maturation antigen (BCMA) (NCT04650724) is feasible. Taken together of the contemporary literature, the promising results on the effect of anti-BCMA CAR-T cell therapy and allogeneic HSCT might present a proof-of-principle for patients with EMM, and therefore, patients with the disease need to be included in future studies.

## Introduction

Extramedullary multiple myeloma (EMM) is an aggressive sub-entity of multiple myeloma (MM). Importantly, the definition of EMM should refer to purely extramedullary disease and so explicitly exclude “solitary extramedullary plasmacytoma” and “bone-related plasmacytomas arising from the neighboring bone marrow” (1, 2). EMM is found in 6–8% of newly diagnosed MM (NDMM) patients, and the prevalence of EMM increases during the disease course with 10–30% of patients (3). Despite an excellent improvement in survival for most patients with MM over recent decades, the outcomes are generally dismal when EMM develops. The overall survival (OS) of patients with EMM in various informative studies was usually not longer than 3 years (2), especially for patients refractory to standard therapies or relapse after autologous hematopoietic stem cell transplantation (ASCT) with a median OS of <1 year (4). Standard treatment for EMM has not been established. Although, most patients with MM respond to modern first-line therapy, current therapies have not sufficiently improved patient outcomes with EMM. Several studies suggested that regimens containing bortezomib and/or immunomodulatory drugs (IMiDs) improved outcomes; however, the gains in progression-free survival (PFS) and OS were less pronounced than those in the case of classic MM (5, 6). Furthermore, most studies consistently showed an inferior outcome of patients with EMM despite the use of ASCT. Even regarding the efficacy of daratumumab, a CD38-targeting antibody, either alone or in combination, the overall response rate was only 16.7% in EMM (7), with a median PFS and OS of 2.3 and 6.6 months, respectively (8). Therefore, innovative strategies are critically needed. This study presented a heavily pretreated patient with relapsed EMM and refractoriness to a proteasome inhibitor (PI; bortezomib), a next-generation PI (ixazomib), IMiDs (lenalidomide), ASCT, and monoclonal antibody (directed against CD38: daratumumab) and indicated that myeloablative haploidentical hematopoietic stem cell transplantation (haploidentical-HSCT) as a salvage treatment of relapse after a chimeric antigen receptor (CAR)-T cell therapy that targeted B-cell maturation antigen (BCMA) is feasible. The study was approved by the Ethics Committee of Sir Run Run Shaw Hospital at Zhejiang University School of Medicine. The study was registered as NCT04650724 at ClinicalTrials.gov. Written informed consent was obtained from the patient for the publication of any potentially identifiable images or data included in this article.

## Case Presentation

**Figure 1:**
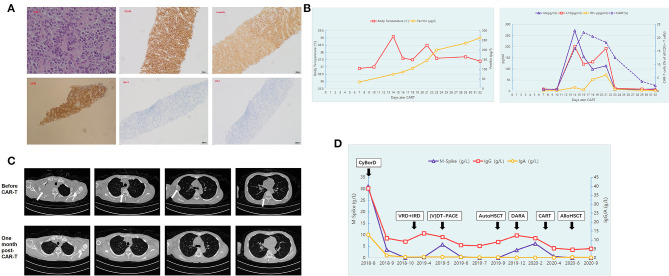
Summary of laboratory findings and clinical course. **(A)** Hematoxylin and eosin staining (original magnification, ×400) and immunohistochemical staining for CD138 (original magnification, ×100), Lambda (original magnification, ×100), and CD38 (original magnification, ×100), BCL-2 (original magnification, ×100) and PD-1 (original magnification, ×100) of the biopsy of the right subpleural extraosseous soft mass. **(B)** The patient presented with fever with the highest temperature at 39°C 14 days post-CAR-T cells infusion, and her serum ferritin level gradually rose to reach a peak level. The trends of serum interleukin (IL)-6, IL-10, and interferon γ (IFN-γ) concentrations and the expansion trend of CAR-T cells are also shown during the course of CAR-T therapy. **(C)** Multiple abnormal masses in the right lung disappeared and could not be detected by CT scanning 1 month post-CAR-T cell therapy. **(D)** The trends of the serum IgA, IgG, and monoclonal protein concentrations (M spike) throughout the treatment.

A 46-year-old female patient was referred to the hospital in August 2018 with a 3-week history of recurrent chest pain. Chest computed tomography (CT) scanning showed a right subpleural extraosseous soft mass (7.6 × 2.06 cm^2^). Subsequent imaging on whole-spine magnetic resonance imaging and X-ray skeletal survey showed multiple bone destruction of the ribs, vertebrae, and pelvis. Laboratory investigations revealed anemia with a hemoglobin level of 86 g/L, hypercalcemia (3.53 mmol/L), and renal dysfunction (serum creatinine 139 μmol/L). The serum IgG level was 38.8 g/L [normal range (NR) < 15.6 g/L], the IgA level was 12.9 g/L (NR <4.53 g/L), and the serum protein electrophoresis showed a paraprotein of 31 g/L. Further serum immunofixation electrophoresis confirmed double clones of IgA and IgG lambda (λ). The amount of serum λ light chain was 2,780 mg/dl (NR <723 mg/dl). A bone marrow aspirate and biopsy demonstrated λ-restricted clonal plasma cell infiltration (43.6%) with a CD138^+^, CD38^+^, CD19^−^, and CD56^+^ phenotype. The albumin level was 26.2 g/L, the beta-2-microglobulin level was 6.0 mg/L, and the lactate dehydrogenase level was within normal limits. Fluorescence *in situ* hybridization confirmed cytogenetics features, including IgH-FGFR3/t (4, 9) translocation, gain of 1q21, and del 13q14. She agreed with the biopsy of the right subpleural extraosseous soft mass. The pathological diagnosis suggested an EMM infiltration, whose genotype was CD38^+^, CD138^+^, Kappa^−^, Lambda^+^, CD20^−^, CD3^−^, Syn^−^, CgA^−^, CK-pan^−^, BCL-2^−^, PD-1^−^, and Ki-67 (80%) (Figure 1A). The patient was diagnosed with double clones of IgA/IgG lambda (λ) –MM with EMM (Durie–Salmon stage IIIA, ISS stage III).

Following 2 cycles of induction chemotherapy with weekly cyclophosphamide, bortezomib, and dexamethasone (CyBorD), the M-band and the right subpleural soft mass could not be detected. After 4 cycles of CyBorD, a CT scan indicated no evidence of abnormal mass in the right lung. No monoclonal band was detected, and the free light chain ratio returned to normal (0.409, upper limit of normal 1.56). She achieved a stringent complete response (sCR) according to the International Myeloma Working Group (IMWG) response criteria. In view of good response, peripheral blood stem cells (PBSCs) were harvested with high dose cyclophosphamide. PBSC harvesting yielded 2.2 × 10^6^/kg CD34-positive stem cells in total.

Unfortunately, when the patient planned to proceed to subsequent ASCT, the right subpleural soft mass recurred with a size of 6.21 × 1.24 cm^2^. The bone marrow examination revealed no clonal plasma cells. The serum and urine protein electrophoresis showed that the amounts of free light chains were within normal limits. The biopsy of the recurred soft mass was performed again, and the pathological genotype was the same as the *de novo* mass. The positron emission tomography-CT (PET-CT) scan indicated pleural thickening with abnormal fluorodeoxyglucose uptake. The patient was switched to second-line treatment with VRD (bortezomib, lenalidomide, and dexamethasone) and IRD (ixazomib, lenalidomide, and dexamethasone). She failed to achieve control with the right subpleural extraosseous soft mass enlarged to 9.53 × 2.33 cm^2^. The patient was treated with three cycles of intensive chemotherapy with the (V) DT-PACE regimen (bortezomib, dexamethasone, thalidomide, cisplatin, doxorubicin, cyclophosphamide, and etoposide) because carfilzomib, pomalidomide, and daratumumab were not available at that time in China. Consequently, the soft mass reduced in size and then disappeared and hence could not be detected by CT scanning. In September 2019, ASCT was performed after conditioning with melphalan (140 mg/m^2^) and bortezomib (1 mg/m^2^ four times). Thereafter, the right subpleural soft mass could no longer be detected. The patient received maintenance therapy with lenalidomide and bortezomib after transplantation.

However, in December 2019, 3 months after ASCT, the size of the subpleural soft mass increased again. Although, the serum monoclonal protein concentration (M-band) reached 3.32 g/L, no clonal plasma cells were detected on bone marrow examination. She received a weekly regimen with daratumumab, bortezomib, and dexamethasone. This combination therapy, however, failed to achieve any response. The patient developed multiple abnormal masses in the right lung with the M-band increased to 6.16 g/L. The patient developed numbness and pain in the right upper limb and limited movement of the right finger due to nerve compression caused by the rapid progression of lung mass. Considering her poor prognosis, the patient was enrolled in a BCMA-targeted CAR-T trial in our center (NCT04650724). The patient's bone marrow sample obtained at diagnosis showed strong positive BCMA expression (95.85%) on the clonal plasma cells, as detected by flow cytometry. On March 9, 2020, the patient was infused with autologous CAR-T cells targeting BCMA at a dose of 1.0 × 10^6^/kg after conditioning chemotherapy of fludarabine and cyclophosphamide. The patient presented with fever 11 days after infusion of CAR-T cells, and the peak of temperature was 39°C on day 14 and lasted for a total of 16 days. Her serum ferritin level and serum interleukin (IL)-6, IL-10, and interferon γ (IFN-γ) concentrations also gradually rose to reach a peak level during CAR-T cell expansion to peak (Figure 1B). Grade 1 cytokine release syndrome (CRS) was considered according to the guidelines of the CARTOX Working Group (10). Under supportive care without use of tocilizumab or glucocorticoid, the patient's body temperature and the inflammatory cytokines returned into NRs within 14 days. The patient also achieved recovery from cytopenia. Four weeks post-CAR-T cell therapy, repeated CT showed no evidence of a subpleural soft mass, and flow cytometry showed minimal residual disease (MRD) negativity in the bone marrow. The M-band was not detected, and immunofixation electrophoresis was negative. The trends of the size of the subpleural soft mass before and after CAR-T cell therapy detected by CT are shown in Figure 1C.

The disease-free state lasted for 3 months after the infusion of CAR-T cells, and the right subpleural soft mass recurred again with a size of 3.8 × 3.6 cm^2^. In June 2020, the patient underwent HLA-haploidentical HSCT from her son with a myeloablative conditioning regimen consisting of cytarabine [4 g/(m^2^ · d) IV on days −10 to −9], Bu [3.2 mg/(m^2^ · d) IV on days −8 to −6], Cy [1.8 g/(m^2^ · d) IV on days −3 to −2], and rabbit anti-thymocyte globulin (Genzyme, MA, USA) (7.5 mg/kg total dose). The HLA-haploidentical HSCT was tolerated well with grade 1 mucositis and an episode of fever during the neutropenic phase, managed at the ward level with antimicrobials and supportive care. Another adverse event during the treatment was grade 1 nausea. Complete hematological response and sCR were achieved by day 30 after HSCT. At the moment of writing, the patient was in good clinical condition and had a good performance status. She is currently in a state of sCR with complete donor chimerism. The trends of the serum IgA, IgG, and monoclonal protein concentrations (M spike) throughout the treatment are shown in Figure 1D.

## Discussion

Advancements in treatment, including the introduction of IMiDs, PIs, and monoclonal antibodies, have prolonged the survival of patients with MM. However, nearly all patients, even those who achieve a complete response, inevitably relapse or become refractory to therapy. A multicenter IMWG study showed that patients with refractory relapsed MM (RRMM), who received at least three prior lines of therapy, were refractory to both an IMiD (lenalidomide or pomalidomide) and a PI (bortezomib or carfilzomib), and were exposed to an alkylating agent, had a median OS of 13 months (11). Daratumumab is a CD38-targeting monoclonal antibody (CD38 MoAB) with remarkable activity in RRMM. Daratumumab monotherapy in heavily pretreated patients with RRMM had an overall response rate of 31% with a median OS of 20.1 months (7). However, patients with MM refractory to CD38 MoAB had a dismal prognosis with a median OS of only 8.6 months (12). Hence, it is clear that new therapeutic approaches need to be developed to further prolong disease control beyond what is afforded by the currently available drugs. The introduction of CAR-modified T cells has revolutionized immunotherapy and cancer treatment as a whole. To date, anti-BCMA CAR-T cells have shown remarkable results in published clinical trials. Data from 18 published phase I/II clinical trials encompassing nearly 300 patients with MM treated with anti-BCMA CAR-T cells showed that the overall response rate was about 64–100% with acceptable rates of grades 3–4 CRS and neurotoxicity (13). Moreover, the efficacy of CAR-T cell therapy was not significantly influenced by previous treatment exposure. In a phase I study involving 33 patients with RRMM, in which 79% of patients were exposed to bortezomib, carfilzomib, lenalidomide, pomalidomide, and daratumumab, the objective response rate of anti-BCMA CAR-T cell therapy was 85%, including 15 patients (45%) with a complete response (14).

However, the outcomes remain particularly poor when patients with MM develop EMM. The currently available standard therapies have not sufficiently improved the outcomes for patients with EMM. To the best of our knowledge, there are no prospective clinical trials that have been specifically dedicated to EMM patients. Therefore, it is difficult to recommend a specific treatment strategy over another. CAR-T cell therapy, tandem ASCT, and novel antibodies have recently shown promising results in a limited number of patients with EMM. Herein, the clinical data of these agents are summarized in Table 1.

**Table 1:** Outcomes of patients with EMM after treatment with CAR-T cell therapy, novel agents, and stem cell transplant.

**Number of MM patients (period covered)**	**Incidence of EMM, *n* (%)**	**Prior therapy**	**Treatment plan**	**ORR, OS, or PFS**	**Reference**
**CAR-T cell therapy**					
17 (April to November 2017)	5 (29.4%)	Bortezomib and lenalidomide for all patients including one patient also receiving carfilzomib, ixazomib, pomalidomide, and ASCT	Anti-BCMA CAR-T (LCAR-B38M)	ORR: 90%	Xu et al. (15)
33 (January 2016 to April 2018)	9 (27%)	Bortezomib, carfilzomib, lenalidomide, pomalidomide, and daratumumab	Anti-BCMA CAR-T (bb2121)	ORR: 89%	Raje et al. (14)
25 (November 2015 to December 2017)	7 (28%)	Bortezomib, carfilzomib, lenalidomide, pomalidomide, daratumumab, and ASCT	Anti-BCMA CAR-T	ORR: 57%	Cohen et al. (16)
**Novel agents**					
196 (June 2018 to January 2019)	40 (20.4%)	Bortezomib, carfilzomib, lenalidomide, pomalidomide, and anti-CD38 monoclonal antibodies (daratumumab, isatuximab)	Belantamab mafodotin (GSK2857916) (an immunoconjugate targeting BCMA), with 2.5 or 3.4 mg/kg	ORR: 9.1% in 2.5 mg/kg cohort, 5.6% in 3.4 mg/kg cohort	Lonial et al. (17)
157 (December 2016 to October 2019)	55 (35%)	Anti-CD38 monoclonal antibodies (daratumumab or others), pomalidomide, proteasome inhibitor, and alkylator therapy	Melflufen 40 mg intravenously on day 1 of each 28-day cycle plus once weekly oral dexamethasone at a dose of 40 mg (20 mg in patients older than 75 years)	The ORR was 29% in the all-treated population, with 26% in the triple-class-refractory population. In the all-treated population, the median duration of response was 5.5 months, median PFS was 4.2 months, and median OS was 11.6 months at a median follow-up of 14 months. Of patients in the extramedullary subgroup, 13 patients achieved a PR or better as the best response.	Richardson et al. (18)
**ASCT or allo-HSCT**					
226 (January 2010 to November 2017)	176 patients (77.9%) with EMM and 50 patients with bone-related plasmacytomas	Bortezomib, carfilzomib, thalidomide, lenalidomide, and pomalidomide	100 patients (44.2%) receiving ASCT	The median PFS was 49 vs. 28.1 months in patients undergoing ASCT compared with those who did not (*P* < 0.001).	Beksac et al. (19)
3,744 adult patients with NDMM (2005–2014)	139 (3.7%)	Not reported	Up-front single ASCT (*n* = 124), tandem ASCT (*n* = 15)	Patients with EMM involvement showed no significant difference in both 3-year PFS and OS after tandem vs. single transplantation: 56.2 (95% CI: 27.2–85.3) vs. 48.3% (95% CI: 36.6–60.1; *P* = 0.98) and 52.0 (95% CI: 20.0–84.0) vs. 64.9% (95% CI: 54.2–75.7; *P* = 0.39).	Gagelmann et al. (20)
488 adult patients with NDMM and extramedullary involvement (2003–2015)	488 (100%)	355 patients (73%) receiving bortezomib-based induction therapy and 133 patients (27%) receiving non-bortezomib-based induction therapy before first transplant	Single ASCT (*n* = 373), tandem ASCT (*n* = 84), or auto–allo HSCT (*n* = 31)	Tandem ASCT was significantly associated with better OS (*P* = 0.02) and PFS (*P* = 0.03) than single ASCT, whereas auto–allo HSCT did not show the survival advantage compared with single ASCT or tandem ASCT	Gagelmann et al. (21)

Data regarding the efficacy of newer classes of drugs, such as carfilzomib, daratumumab, and so on, either alone or in combination, in EMM are limited. Published data suggested that the presence of EMM resulted in a strong trend toward a shorter duration of response than that of classic MM despite treatment with novel drugs (8, 9). In a study to analyze the efficacy of pomalidomide and dexamethasone in 21 patients from 9 hospitals of Catalonia (Spain), with relapsed or refractory MM with paraskeletal plasmacytomas or EMM, there were no responses observed among patients with EMM (22). Recently, an open-label, two-arm, phase 2 study at 58 MM specialty centers in 8 countries was done to investigate the safety and activity of belantamab mafodotin (GSK2857916), an immunoconjugate targeting BCMA, in 196 adult patients with relapsed or refractory MM with disease progression after three or more lines of therapy and who were refractory to IMiDs and PIs and refractory or intolerant (or both) to an anti-CD38 monoclonal antibody. Forty of 196 patients have EMM and achieved an overall response of 9.1% when receiving 2.5 mg/kg of GSK2857916 and 5.6% when receiving 3.4 mg/kg. However, the overall response rates were 31% and 34% in patients without EMM, respectively (17). Richardson recently reported the results of the phase II HORIZON trial with melphalan flufenamide (melflufen) in RRMM, which is a first-in-class peptide–drug conjugate that targets aminopeptidases and rapidly and selectively releases alkylating agents into tumor cells. Of 157 patients with RRMM refractory to pomalidomide and/or an anti-CD38 monoclonal antibody enrolled and treated, 55 (35%) had EMM. Of patients in the extramedullary subgroup, 13 patients achieved a partial remission (PR) or better as the best response to melflufen plus dexamethasone (18).

Emerging cellular therapies, including CAR-T cell therapy, also hold promise to improve the prognosis of EMM. However, data reporting the efficacy of CAR-T cell therapy in EMM are scarce. In clinical trials of anti-BCMA CAR-T cell therapy with available data on patients with extramedullary disease or not, the response rates of 57–90% were observed among patients who had EMM at baseline (14–16) (Table 1). Further, prospective studies of CAR-T cell therapy should be designed for MM patients with EMM.

Few studies suggested that ASCT could overcome the poor prognostic impact of EMM. In a retrospective multi-institutional study from Europe, 100 patients with EMM underwent ASCT, of which 51.5% had primary EMM. Patients undergoing ASCT achieved a superior PFS than those who did not undergo ASCT (median PFS: 49 vs. 28.1 months, *P* < 0.001) (19). However, most studies consistently showed an inferior outcome despite the use of ASCT (2). With respect to efficacy of tandem ASCT, a study from the Chronic Malignancies Working Party of the European Society for Blood and Marrow Transplantation (EBMT) including 3,744 adult MM patients who received up-front single (*n* = 3,391) or tandem ASCT (*n* = 353) between 2005 and 2014 with available data on extramedullary involvement at diagnosis, data showed that first-line treatment with tandem ASCT compared with single transplantation resulted in similar survival in patients with extramedullary disease at diagnosis (*P* = 0.13) (20). Thereafter, the EBMT further conducted a study to analyze data from 488 adult MM patients with EMM, who underwent single ASCT (*n* = 373), tandem ASCT (*n* = 84), or autologous–allogeneic transplant (*n* = 31) between 2003 and 2015. In multivariate analysis, tandem ASCT significantly improved OS and PFS vs. single ASCT and may overcome the poor prognosis of high-risk cytogenetics. However, autologous–allogeneic transplant did not significantly differ in outcomes but appeared to improve OS, but results were limited because of the small population (21) (Table 1).

Allogeneic hematopoietic cell transplantation (allo-HCT) is one of the most promising ways of restoring the immune system's ability to recognize and destroy MM cells and is currently the only potentially curative approach for patients with MM. However, the cumulative incidence of relapse after allo-HCT was significantly lower; this therapeutic modality was severely hampered by the high treatment-related morbidity and mortality (23, 24). Allo-HSCT might benefit carefully selected patients with a median OS of 39.2 months; the non-relapse mortality (NRM) was relatively low with a cumulative incidence of 12.4% after 10 years (25). Few studies explored the efficacy of HLA-haploidentical HSCT in MM. A multicenter investigation from China compared the main outcomes of haploidentical HSCT in patients with MM with the outcomes of HLA-matched sibling HSCT. The data showed no statistically significant differences in relapse, NRM, PFS, and OS between the two groups (26). A report from the EBMT/Center for International Blood and Marrow Transplant Research (CIBMTR) suggested that haploidentical HSCT was feasible for patients with multiply relapsed or high-risk MM, with an encouraging 2-year OS of 48% and an NRM of 21% at 1 year, supporting further investigation of haploidentical HSCT in suitable candidates with MM (27).

Although, in some patients, CAR-T cell therapy can induce sustained remission and replace allo-HSCT. CAR-T cells combined with HSCT may be a more effective strategy to decrease the risk of relapse. Clinical trials are now needed to address the relative roles of CAR-T cells and HSCT in the context of transplantation-eligible patients. Anti-CD19 CAR-T therapy as consolidation therapy after high-dose melphalan and autologous HSCT has been used to a patient with refractory MM at the University of Pennsylvania, which led to a CR with no evidence of progression and no measurable serum or urine monoclonal protein at 12 months after treatment (28). As one way to prevent relapse, CAR-T cells can induce remissions as a bridge to allo-HSCT in B-cell malignancies (29). Our patient relapsed after anti-BCMA CAR-T cell therapy and was rescued by a haploidentical HSCT. The follow-up after haploidentical HSCT to our case is short and needs longer follow-up to confirm the efficiency of haploidentical HSCT to maintain remission. Transplantation-related mortality, such as graft-versus-host disease (GVHD), should also be paid attention to. We can only conclude that myeloablative haploidentical HSCT as a salvage treatment of relapse after anti-BCMA CAR-T cell therapy is feasible. Although anti-BCMA CAR-T cell therapy provides very high rate of response in RRMM, most of the studies still show PFS less than a year. Maintenance regimens post-CAR-T cell therapy, such as subsequently planned allo-HSCT or subsequent multiple CAR-T cell infusions over time, or maintenance treatment with PIs and/or IMiDs should be considered as some of the future directions.

In conclusion, the present case study highlighted the poor outcome for patients with relapsed refractory EMM. The findings indicated that these patients needed more specific and less toxic ways of exploiting the myeloma-targeting capabilities of the immune system. In the future, subgroup analyses of large prospective trials focusing on EMM should be conducted to address this issue.

## Data Availability Statement

The datasets presented in this article are not readily available. Requests to access the datasets should be directed to haowenxiaoxiao@zju.edu.cn.

## Ethics Statement

The studies involving human participants were reviewed and approved by the study was approved by the Ethics Committee of Sir Run Run Shaw Hospital at Zhejiang University School of Medicine. The patients/participants provided their written informed consent to participate in this study. Written informed consent was obtained from the individual(s) for the publication of any potentially identifiable images or data included in this article.

## Author Contributions

HX, YQ, and LY designed the research study. HX, ZQ, and XZ analyzed the data and wrote the paper. HM and LY provided the CAR-T cells and performed the related experiments. YQ, XZ, WP, and HX treated and managed the patient. All authors contributed to the article and approved the submitted version.

## Conflict of Interest

HM and LY were employed by the company PersonGen BioTherapeutics (Suzhou) Co., Ltd. The remaining authors declare that the research was conducted in the absence of any commercial or financial relationships that could be construed as a potential conflict of interest.
