# Supplementary figures and images for: Successful Treatment of Relapsed/Refractory Extramedullary Multiple Myeloma With Anti-BCMA CAR-T Cell Therapy Followed by Haploidentical Hematopoietic Stem Cell Transplantation: A Case Report and a Review of the Contemporary Literature
Source: Front Med (Lausanne). 2021 May 7;8:649824. doi: 10.3389/fmed.2021.649824 (PMC8138324; doi:10.3389/fmed.2021.649824)

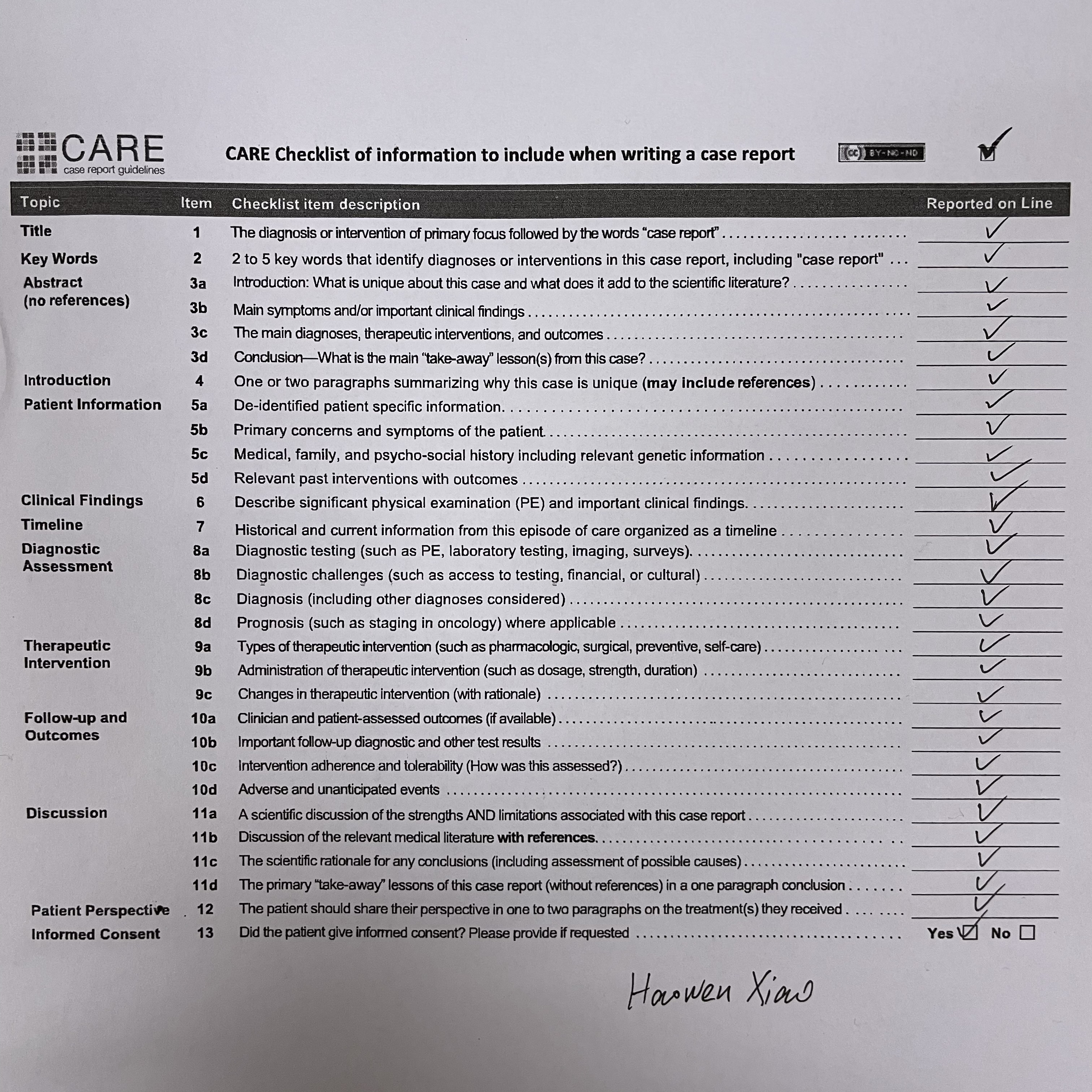

Supplement: Supplementary file 1 [file Image_1.JPEG]
